# WHO Collaborating Centres: a global scientific network

**DOI:** 10.2471/BLT.26.296055

**Published:** 2026-04-01

**Authors:** Meg Doherty, Matias Tuler, Sylvie Briand, Tedros Adhanom Ghebreyesus

**Affiliations:** a Science Division, World Health Organization, Avenue Appia 20, 1211 Geneva, Switzerland.; b World Health Organization, Geneva, Switzerland.

Every year on 7 April, the world celebrates World Health Day,^1^ commemorating the date the Constitution of the World Health Organization (WHO)^2^ entered into force in 1948. This year, WHO has chosen the theme “*Together for health. Stand with science”* to highlight the role of science-led innovations in improving global public health and to reinforce evidence-driven action in a multilateral world.

The Constitution establishes that one of the functions of WHO is to promote and conduct research in the field of health. The practice of relying on national institutions to serve international functions started under the League of Nations, when national laboratories were selected as reference centres responsible for standardizing biological products. The newly formed WHO expanded this approach by designating additional reference centres. The first was the World Influenza Centre^3^ in London, United Kingdom of Great Britain and Northern Ireland, established in 1948 to conduct global epidemiological surveillance.

In 1949, the Second World Health Assembly adopted a policy that has been consistently applied since, stating that WHO should not establish its own research institutions but support, coordinate and use the work of existing institutions around the world.^4^ This approach was the framework for what years later became the WHO Collaborating Centres.

A collaborating centre is defined as an institution officially designated by the WHO Director-General to support WHO programmes at national, regional, interregional and global levels.^5^ As of 2026, over 800 collaborating centres at universities, hospitals and large research institutes in over 80 Member States^5^ support WHO to deliver on its mandated work. These national scientific institutions represent the largest scientific network connected to any United Nations agency and is increasingly seen as a resource for a global movement to promote science, multilateralism, evidence and trust.

In keeping with WHO’s strategy for technical cooperation, these centres also reinforce national health capacity by contributing information, services, research and training to support improvements in public health. This collaboration mechanism has considerably strengthened the participation of national institutions in WHO programmes.

All areas of WHO’s work benefit from the collaborating centres, whose activities are varied: gathering, analysing and sharing data; standardizing terminology, technology, diagnostics and therapeutics. Centres provide reference materials; participate in collaborative research under WHO leadership; provide training; and coordinate work conducted by multiple institutions on specific topics.

Successful collaborations result in a win-win situation for both parties: leading institutions support WHO in achieving its mandated functions by providing expertise. These collaborations ensure the scientific credibility of global health initiatives and strengthen WHO’s leadership role in shaping the international health agenda. Conversely, the designation as a WHO collaborating centre brings recognition and visibility among national authorities and the public, facilitates information exchange, enhances opportunities for international technical cooperation, and in some cases also helps attract additional funding from partners.

Designation is not linked to financial support from WHO. Instead, the designated institutions are normally responsible for covering the costs of work they do at the request of WHO and in support of WHO’s programmes. This model allows WHO to access resources that would otherwise not be accessible. Given the large number of collaborating centres, this mechanism of collaboration has become the largest in-kind resource mobilization modality supporting WHO priorities and activities.

WHO has brought together collaborating centres working in similar technical areas to form technical, regional and national networks that further optimize the breadth and impact of their work with WHO, for example on influenza, bioethics, radiation, children’s environmental health^6^ and classification of diseases. 

The geopolitical rupture that has unfolded over the past year has presented WHO with several challenges and opportunities, and triggered a process of prioritization and realignment in WHO that has prompted us to examine how, as a leaner Organization, we can be more effective, including by leveraging the network of collaborating centres and the in-kind support they provide for global health impact.

On World Health Day, WHO will convene its collaborating centres to celebrate their achievements and forge a path for greater and stronger collaboration at the first Global Forum of WHO Collaborating Centres.^7^ This collaborative model has stood the test of time for nearly 80 years. In a time of political instability, changing global health architecture, reduced health funding and an erosion of confidence in science, WHO will continue to rely on the WHO Collaborating Centres. The Organization aims to expand these collaborations to leverage the good will of researchers and scientists worldwide to contribute to WHO’s goal of achieving health for all and to keep standing with science.
